# An LRP1-binding motif in cellular prion protein replicates cell-signaling activities of the full-length protein

**DOI:** 10.1172/jci.insight.170121

**Published:** 2023-08-08

**Authors:** Elisabetta Mantuano, Carlotta Zampieri, Pardis Azmoon, Cory B. Gunner, Kyle R. Heye, Steven L. Gonias

**Affiliations:** Department of Pathology, University of California San Diego School of Medicine, La Jolla, California, USA.

**Keywords:** Cell Biology, Inflammation, Peptides, Prions, Signal transduction

## Abstract

Low-density lipoprotein receptor-related protein-1 (LRP1) functions as a receptor for nonpathogenic cellular prion protein (PrP^C^), which is released from cells by ADAM (a disintegrin and metalloproteinase domain) proteases or in extracellular vesicles. This interaction activates cell signaling and attenuates inflammatory responses. We screened 14-mer PrP^C^-derived peptides and identified a putative LRP1 recognition motif in the PrP^C^ sequence spanning residues 98–111. A synthetic peptide (P3) corresponding to this region replicated the cell-signaling and biological activities of full-length shed PrP^C^. P3 blocked LPS-elicited cytokine expression in macrophages and microglia and rescued the heightened sensitivity to LPS in mice in which the PrP^C^ gene (*Prnp*) had been deleted. P3 activated ERK1/2 and induced neurite outgrowth in PC12 cells. The response to P3 required LRP1 and the NMDA receptor and was blocked by the PrP^C^-specific antibody, POM2. P3 has Lys residues, which are typically necessary for LRP1 binding. Converting Lys^100^ and Lys^103^ into Ala eliminated the activity of P3, suggesting that these residues are essential in the LRP1-binding motif. A P3 derivative in which Lys^105^ and Lys^109^ were converted into Ala retained activity. We conclude that the biological activities of shed PrP^C^, attributed to interaction with LRP1, are retained in synthetic peptides, which may be templates for therapeutics development.

## Graphical abstract

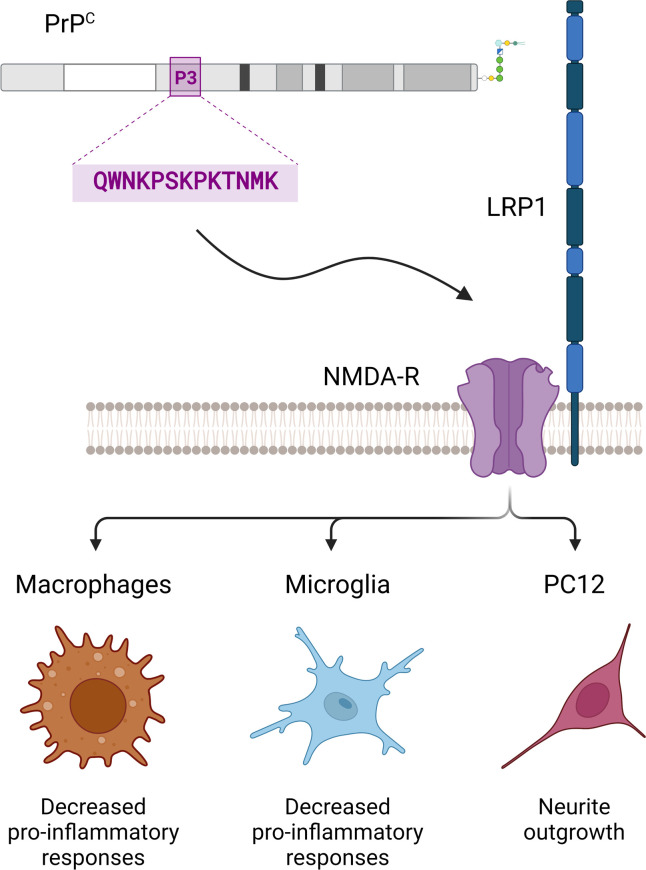

## Introduction

Low-density lipoprotein receptor-related protein-1 (LRP1) is a type 1 transmembrane protein that functions as an endocytic and cell-signaling receptor for diverse ligands, including lipoproteins, proteases, protease inhibitors, growth factors, extracellular matrix proteins, heat shock proteins, and proteins released by injured and dying cells, including microtubule-associated protein tau and α-synuclein (1–5). The evolutionary foundation for a receptor with such a broad scope of ligands remains unclear; however, LRP1 may function as an injury detection receptor, as has been most fully defined for Schwann cell LRP1 (6–8). Specificity in the function of LRP1 may be manifested in the ability of different ligands to elicit diverse cell-signaling responses by engaging distinct cell-signaling coreceptors, including but not limited to the NMDA receptor (NMDA-R), tropomyosin receptor kinase receptors, and the p75 neurotrophic receptor (9–15).

Nonpathogenic cellular prion protein (PrP^C^) is expressed by numerous cells inside and outside the nervous system (16, 17) and interacts with LRP1 in 3 states. First, PrP^C^, which is glycosylphosphatidylinositol (GPI) anchored to the plasma membrane, laterally associates with LRP1 in the same cell (18–20). Second, PrP^C^, which is released from the cell surface by ADAM (a disintegrin and metalloproteinase domain) proteases, binds to LRP1 (21). Finally, PrP^C^ that is embedded in exosomes and other extracellular vesicles (EVs) associates with LRP1 in target cells (22, 23). We demonstrated that a recombinant derivative of PrP^C^ (S-PrP), corresponding closely to the product released from cells by ADAM10 (24), and PrP^C^-bearing EVs isolated from human plasma activate cell signaling in macrophages and PC12 cells, in an LRP1- and NMDA-R–dependent manner (21–23, 25). As a result, these PrP^C^ derivatives oppose the activity of pattern recognition receptors, including Toll-like receptors, in macrophages and promote neurite outgrowth in PC12 cells.

Molecular analysis of the interaction of LRP1 with a number of ligands, including activated α_2_-macroglobulin (α_2_M), plasminogen activator inhibitor-1, coagulation factor VIII, and receptor-associated protein, has demonstrated an essential role for ligand-associated Lys residues, typically in tandem (26–31). In this study, we screened a series of synthetic peptides, corresponding to the structure of PrP^C^, and identified a putative LRP1-binding motif just distal to the octarepeat region, in the disordered N-terminal half of PrP^C^. A 14-mer synthetic peptide corresponding to the putative LRP1-binding motif (P3) replicated all the cell-signaling activities of full-length S-PrP in a manner that required LRP1 and the NMDA-R. P3 also rescued the increased susceptibility of mice with PrP^C^ gene (*Prnp*) knockout to LPS. Lys^100^ and Lys^103^, from the structure of PrP^C^, were essential for the cell-signaling activity of P3; when both residues were converted to Ala, cell signaling and biological activity were completely eliminated.

Synthetic peptides have been transformed into therapeutics at an increasing rate in recent decades (32). Although PrP^C^ was previously reported to demonstrate antiinflammatory activity in a variety of contexts, including in experimental autoimmune encephalitis (33–35) and in ischemic brain injury (33, 36–38), incomplete understanding of the responsible molecular mechanism has hindered efforts to exploit this activity of PrP^C^ in therapeutics development. The results reported here, identifying P3 and the LRP1/NMDA-R assembly as members of a single system with antiinflammatory activity, set the framework for new studies exploring the efficacy of PrP^C^ derivatives as candidate therapeutics in a variety of disease states in which inflammation plays an important role.

## Results

### A synthetic peptide corresponding to a sequence in the unstructured N-terminal region of PrP^C^ replicates the effects of S-PrP on macrophage physiology.

**Figure 1:**
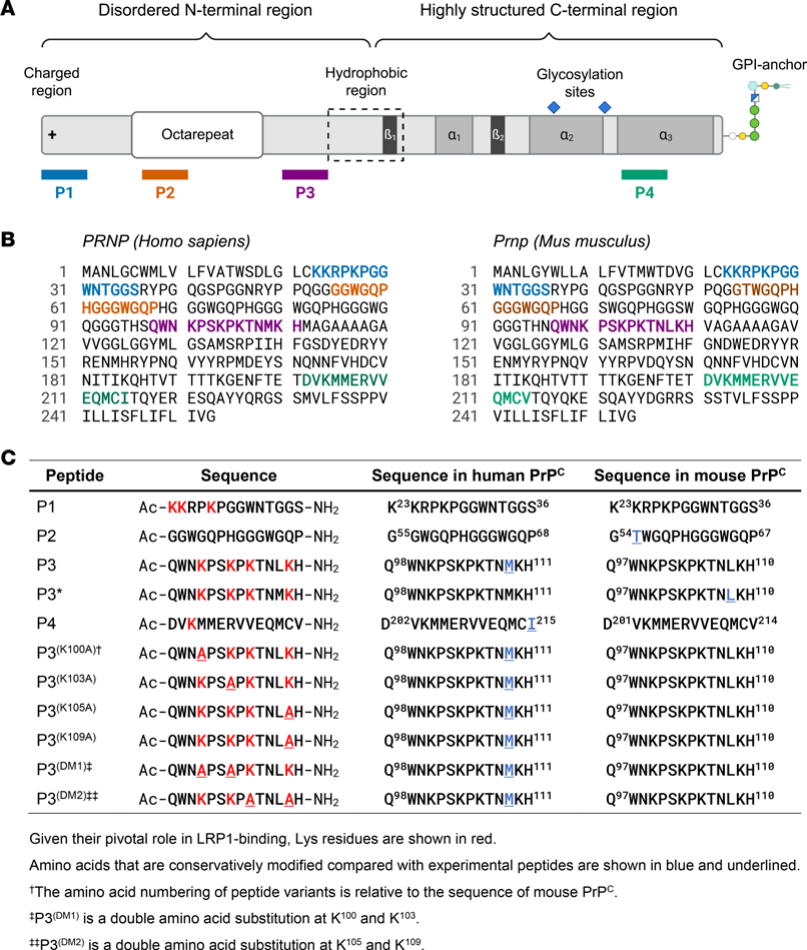
Synthetic peptides and their relation to the structure of PrP^C^. (**A**) Location of the primary set of 4 synthetic peptides in relation to the structure of PrP^C^. (**B**) Using the same color-coding system applied in **A**, P1–P4 are located within the primary sequences of human and mouse PrP^C^. (**C**) The sequences of all studied synthetic peptides, including variants of P3/P3*, are shown. Lys residues and Lys residues that were converted to Ala in second-generation peptides are shown in red. Conservative sequence differences between the synthetic peptides and the structure of human and mouse PrP^C^ are shown in blue and underlined.

We synthesized a series of peptides corresponding to sequences in the structure of PrP^C^, including 2 with clusters of Lys residues (P1 and P3) and 2 without sequence elements suggestive of LRP1 binding (P2 and P4). Three of the peptides corresponded to sequences in the disordered N-terminal region of PrP^C^ (Figure 1A). Differences in the sequences of human and mouse PrP^C^ in the regions corresponding to the synthetic peptides were conservative (Figure 1B). Because P3 emerged as important for the activities studied here, we synthesized 2 variants of this peptide (P3 and P3*) to completely replicate the mouse and human sequences. Figure 1C summarizes the sequences of the first 5 peptides, in relation to the structure of human and mouse PrP^C^, and a secondary set of peptides designed to explore the molecular requirements for engaging the LRP1/NMDA-R assembly.

**Figure 2:**
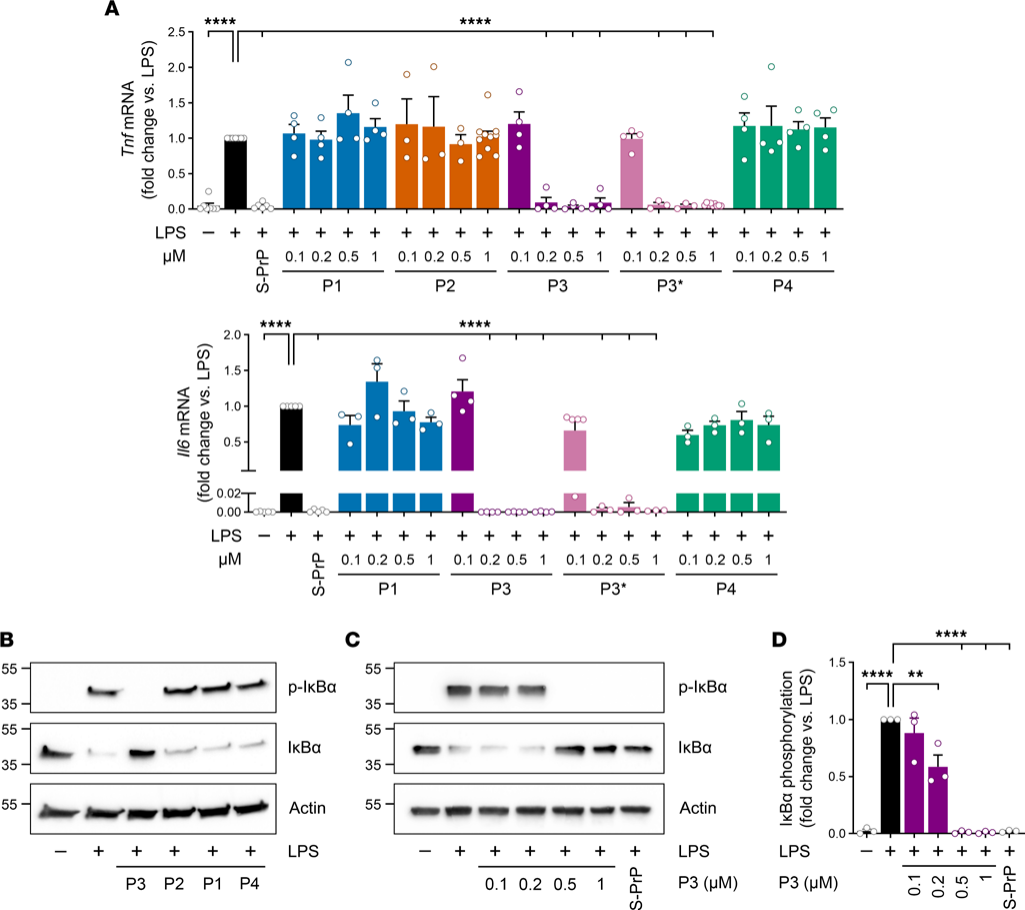
P3 replicates the effects of S-PrP and EV-associated PrP^C^ in macrophages. (**A**) BMDMs from C57BL/6J mice were treated for 6 hours with LPS (0.1 μg/mL) in the presence or absence of S-PrP (40 nM) or increasing concentrations (0.1–1.0 μM) of P1, P2, P3, P3*, or P4. RT-qPCR was performed to determine mRNA levels of *Tnf* and *Il6* (mean ± SEM, *n* = 3–9, individual points shown; 1-way ANOVA: ****P < 0.0001). (**B**) BMDMs were treated for 1 hour with LPS (0.1 μg/mL) in the presence or absence of P1, P2, P3, or P4 (each at 0.5 μM). Immunoblot analysis was performed to detect phosphorylated IκBα, total IκBα, and β-actin. (**C**) BMDMs were treated for 1 hour with LPS (0.1 μg/mL) in the presence or absence of S-PrP (40 nM) or increasing concentrations of P3 (0.1–1 μM). Immunoblot analysis was performed to detect phosphorylated IκBα, total IκBα, and β-actin. (**D**) Densitometry analysis of phosphorylated IκBα band intensity relative to β-actin for cells treated with LPS and different concentrations of P3 (mean ± SEM, *n* = 3, 1-way ANOVA: **P < 0.01, ****P < 0.0001).

Initially, we screened for the ability of PrP^C^-derived peptides to inhibit expression of *Tnf* mRNA, which encodes TNF-α, in response to LPS in bone marrow–derived macrophages (BMDMs) and, thus, replicate the activity of S-PrP and EV-associated PrP^C^ (22, 25). BMDMs were harvested as previously described (39, 40) and treated with 0.1 μg/mL LPS in the presence of increasing concentrations of each peptide for 6 hours. Figure 2A shows that, in the absence of peptides, LPS significantly increased *Tnf* mRNA expression in the BMDMs, as determined by reverse transcription quantitative PCR (RT-qPCR). S-PrP (40 nM) blocked this response, as previously demonstrated (22). P1, P2, and P4 had no effect on LPS-induced *Tnf* expression. By contrast, P3 and P3*, at concentrations of 0.2 μM or higher, completely inhibited LPS-induced *Tnf* expression. S-PrP, P3, and P3* also blocked LPS-induced expression of *Il6*, which encodes IL-6, whereas P1 and P4 were inactive.

Increased expression of pro-inflammatory cytokines in response to LPS requires NF-κB activation, which may be monitored by examining IκBα phosphorylation and the accompanying decrease in total abundance of IκBα (41). Figure 2B shows that in the absence of PrP^C^-derived peptides, BMDMs treated with LPS (0.1 μg/mL) for 1 hour demonstrated increased phosphorylated (p-) IκBα and decreased total IκBα, as anticipated. P1, P2, and P4 (each at 0.5 μM) had no effect on this response. By contrast, 0.5 μM P3 blocked LPS-induced IκBα phosphorylation and the associated decrease in total cellular IκBα. Figure 2C shows that the effects of P3 on IκBα phosphorylation were concentration dependent; P3 at concentrations ≥ 0.5 μM completely inhibited this cell-signaling event whereas 0.2 μM P3 typically generated an intermediate effect. In control studies, 40 nM S-PrP blocked IκBα phosphorylation, as anticipated (22). Densitometry analysis of the results of 3 separate experiments is shown in Figure 2D. Overall, these results demonstrate that P3 replicates the activity of S-PrP and EV-associated PrP^C^ as an inhibitor of LPS-induced cytokine expression and NF-κB activation.

### P3 activity in macrophages requires the NMDA-R and LRP1.

LRP1 and the NMDA-R collaborate to mediate cell signaling in response to S-PrP (21, 22, 25). The requirement for the NMDA-R appears to be absolute. In LRP1-deficient cells, signaling is still observed; however, the concentration of S-PrP required to elicit responses is significantly increased, suggesting a model in which LRP1 “captures” S-PrP and then delivers it to the NMDA-R. BMDMs express the NMDA-R (42).

**Figure 3:**
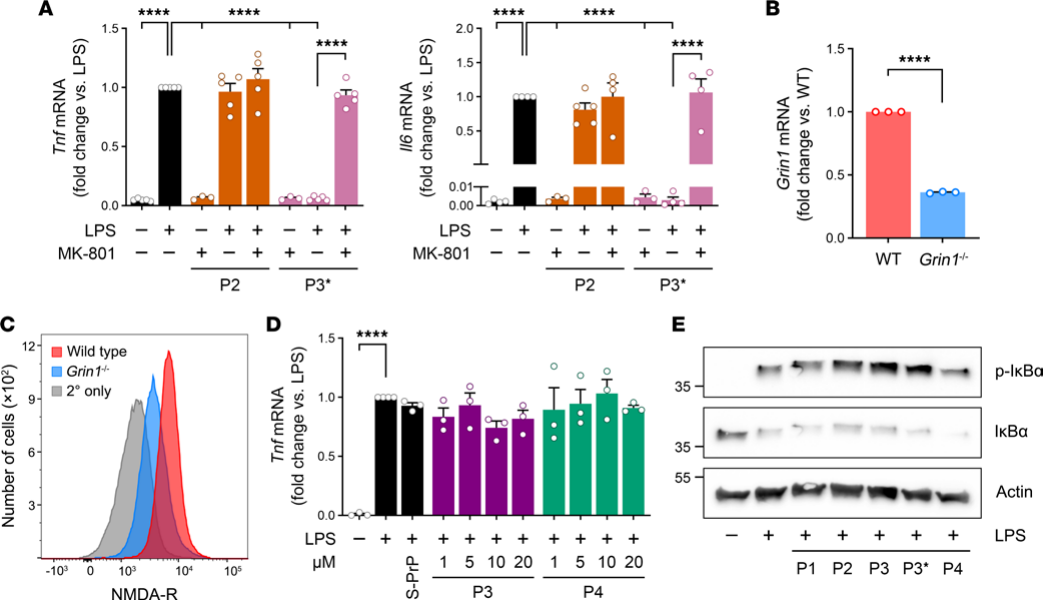
The NMDA-R is necessary for the response to P3 in macrophages. (**A**) BMDMs were pretreated with MK-801 (1 μM) or vehicle for 30 minutes. The cells were then treated with LPS (0.1 μg/mL), P2 (0.5 μM), or P3* (0.5 μM), for 6 hours, as indicated. RT-qPCR was performed to compare mRNA levels for *Tnf* and *Il6* (mean ± SEM, *n* = 3–7, individual points are shown; 1-way ANOVA: ****P < 0.0001). (**B**) BMDMs were harvested from *Grin1*^fl/fl^ LysM-Cre^+^ mice. *Grin1* mRNA expression was determined by RT-qPCR and compared with that detected in BMDMs isolated from *Grin1*^fl/fl^ LysM-Cre^–^ mice (*n* = 3; mean ± SEM; unpaired 2-tailed *t* test: ****P < 0.0001). (**C**) Flow cytometry was performed to detect cell surface GluN1 NMDA-R subunit in BMDMs isolated from *Grin1*^fl/fl^ LysM-Cre–positive and –negative (wild-type) mice. As a control, cells from LysM-Cre^–^ mice were incubated with secondary antibody only (gray). (**D**) BMDMs from *Grin1*^fl/fl^ LysM-Cre^+^ mice were treated for 6 hours with LPS (0.1 μg/mL), in the presence of S-PrP (40 nM) or increasing concentrations of P3 (1–20 μM), P4 (1–20 μM), or vehicle. RT-qPCR was performed to determine *Tnf* mRNA (mean ± SEM, *n* = 3; 1-way ANOVA: ****P < 0.0001). (**E**) BMDMs from *Grin1*^fl/fl^ LysM-Cre^+^ mice were treated for 1 hour with LPS (0.1 μg/mL), in the presence of P1, P2, P3, P3*, and P4, as indicated (each at 0.5 μM). Immunoblot analysis was performed to detect phosphorylated IκBα, total IκBα, and β-actin.

To test whether the NMDA-R is necessary for the response to P3 in macrophages, we treated BMDMs for 6 hours with LPS (0.1 μg/mL) and P2 or P3*, in the presence or absence of the noncompetitive NMDA-R antagonist, MK-801 (Figure 3A). In the absence of MK-801, P3* neutralized the effects of LPS on *Tnf* mRNA expression. P2 was ineffective, as anticipated. In the presence of MK-801, the activity of P3* was blocked, and *Tnf* mRNA expression was restored to the level observed in the absence of P3*. Similarly, MK-801 blocked the ability of P3* to neutralize *Il6* mRNA expression in response to LPS.

To verify a role for macrophage NMDA-R in the response to P3, we bred mice in which the gene encoding the essential NMDA-R GluN1 subunit was floxed (*Grin1*^fl/fl^) with mice that express Cre recombinase under the control of the LysM promoter. BMDMs were harvested from *Grin1*^fl/fl^ LysM-Cre^+^ mice and from control *Grin1*^fl/fl^ LysM-Cre^–^ mice. *Grin1* mRNA was decreased by 63.8% ± 0.4% (*n* = 3) in *Grin1*^fl/fl^ LysM-Cre^+^ BMDMs (Figure 3B). Flow cytometry analysis demonstrated that the abundance of cell surface NMDA-R in *Grin1*^fl/fl^ LysM-Cre^+^ BMDMs was decreased by approximately 70%, as determined by comparing mean fluorescence intensity (Figure 3C).

In GluN1-deficient BMDMs, LPS induced *Tnf* mRNA expression, as anticipated; however, S-PrP (40 nM) failed to inhibit the activity of LPS (Figure 3D). Similarly, P3 was ineffective at inhibiting LPS-stimulated *Tnf* expression, even when the concentration of P3 was increased to 20 μM. None of the PrP^C^-derived peptides (0.5 μM), including P3 and P3*, inhibited LPS-induced IκBα phosphorylation (Figure 3E).

**Figure 4:**
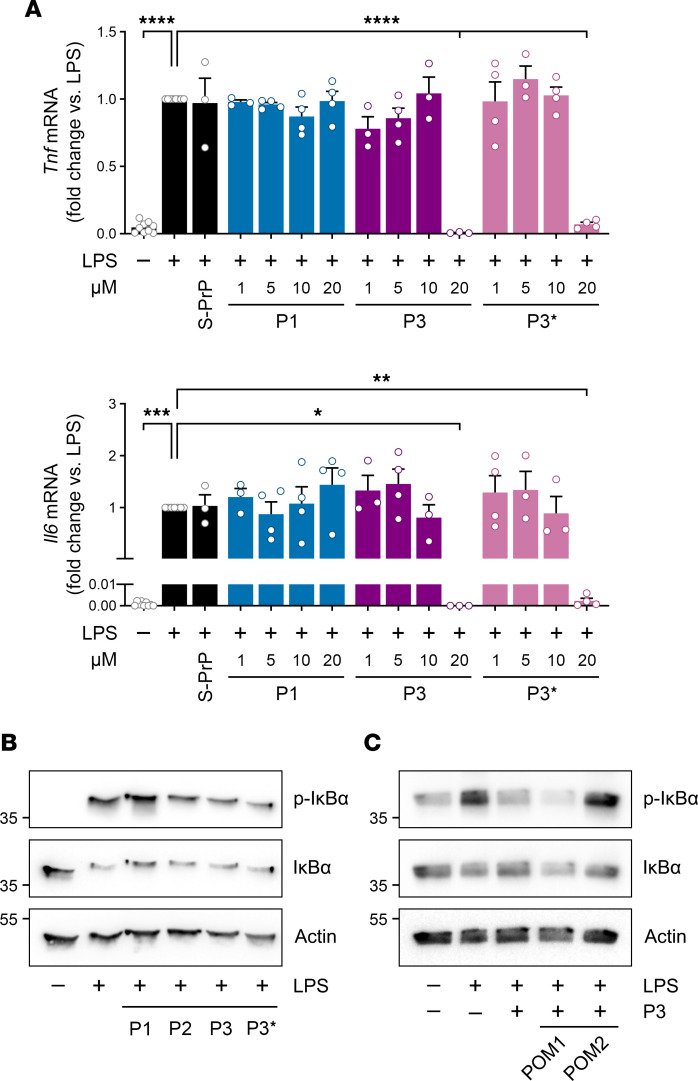
The antiinflammatory activity of P3/P3* is strongly facilitated by LRP1 and blocked by POM2. (**A**) BMDMs from *Lrp1*^fl/fl^ LysM-Cre^+^ mice were treated for 6 hours with LPS (0.1 μg/mL) in the presence of S-PrP (40 nM) or increasing concentrations (1–20 μM) of P1, P3, P3*, or vehicle. RT-qPCR was performed to determine mRNA levels for *Tnf* and *Il6* (mean ± SEM, *n* = 3–4; 1-way ANOVA: *P < 0.05, **P < 0.01, ***P < 0.001, ****P < 0.0001). (**B**) BMDMs were treated for 1 hour with LPS (0.1 μg/mL) in the presence of P1, P2, P3, or P3* (each at 0.5 μM). Immunoblot analysis was performed to detect p-IκBα, total IκBα, and β-actin. (**C**) BMDMs were treated for 1 hour with LPS (0.1 μg/mL) and P3 (0.5 μM), in the presence of POM1 or POM2 (10 μg/mL), as indicated. Immunoblot analysis was performed to detect p-IκBα, total IκBα, and β-actin.

Next, we isolated LRP1-deficient BMDMs from *Lrp1*^fl/fl^ LysM-Cre mice, which were previously described (40). LPS increased expression of *Tnf* mRNA in these BMDMs, as anticipated, and P3 blocked the effects of LPS on *Tnf* expression; however, the minimum concentration of P3 required to inhibit LPS-induced *Tnf* expression was increased about 100-fold to 20 μM (Figure 4A). Equivalent results were obtained with P3*. P1 was inactive as an inhibitor of LPS-induced *Tnf* expression in LRP1-deficient BMDMs throughout the expanded concentration range, as anticipated.

In experiments examining *Il6* mRNA expression, once again P3 and P3* blocked the activity of LPS; however, once again, the minimum concentration of P3 or P3* required to observe activity was increased about 100-fold compared with that observed in wild-type BMDMs. These results mimic those obtained with S-PrP (22) and demonstrate a robust but nonessential role for LRP1 as a facilitator of the activity of P3/P3*. In IκBα phosphorylation experiments using LRP1-deficient BMDMs from *Lrp1*^fl/fl^ LysM-Cre^+^ mice, 0.5 μM P3 and P3* failed to counteract the activity of LPS (Figure 4B), verifying the results of our cytokine mRNA experiments.

In prior studies with S-PrP and EV-associated PrP^C^, we examined a panel of defined PrP^C^-specific monoclonal antibodies (43) and demonstrated that a single antibody from this series, POM2, blocks biological responses mediated by the LRP1/NMDA-R assembly (21–23). Figure 4C shows that POM2 blocked the ability of P3 to antagonize LPS-induced IκBα phosphorylation. POM1 was ineffective in the same studies.

### P3 is bioactive in the PC12 cell culture model system.

**Figure 5:**
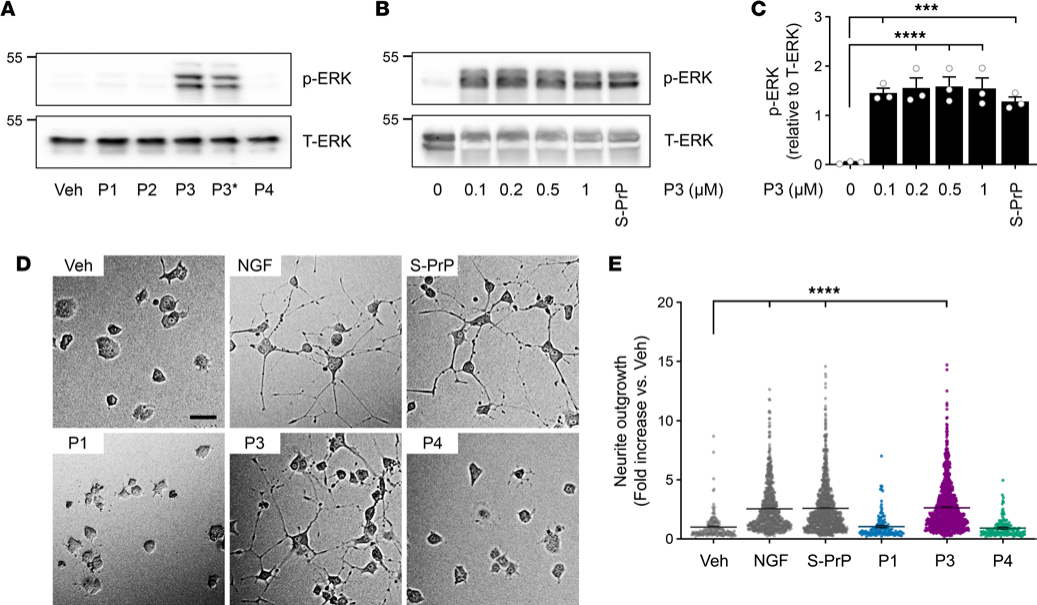
P3 activates cell signaling and promotes neurite outgrowth in PC12 cells. (**A**) PC12 cells were treated with P1, P2, P3, P3*, and P4 (each at 0.5 μM) for 10 minutes. Cell extracts were subjected to immunoblot analysis to detect p-ERK1/2 and total ERK1/2. (**B**) PC12 cells were stimulated for 10 minutes with increasing concentrations of P3 (0.1–1.0 μM) or with S-PrP (40 nM). Phosphorylated ERK1/2 and total ERK1/2 were determined. (**C**) Densitometry analysis of p-ERK1/2 relative to total ERK1/2 (T-ERK) in PC12 cells treated with P3 or S-PrP. The bars represent the mean ± SEM of the results from 3 separate experiments (1-way ANOVA: ***P < 0.001, ****P < 0.0001). (**D**) PC12 cells were treated for 48 hours with S-PrP (40 nM), P1 (0.5 μM), P3 (0.5 μM), P4 (0.5 μM), NGF-β (50 ng/mL) as a positive control, or vehicle. Neurite outgrowth was examined by phase contrast microscopy. Representative images are shown (scale bar, 50 μm). (**E**) Neurite length was determined by analyzing all the cells in ≥5 random fields per treatment, in 3 different experiments (mean ± SEM; 1-way ANOVA: ****P < 0.0001).

S-PrP and EV-associated PrP^C^ activate ERK1/2 and promote neurite outgrowth in PC12 cells (21, 23). We treated PC12 cells with P1, P2, P3, P3*, and P4 (each at 0.5 μM) for 10 minutes. Figure 5A shows that P3 and P3* activated ERK1/2. The other peptides were inactive. ERK1/2 activation by P3 was evident throughout the P3 concentration range studied (0.1–1.0 μM) (Figure 5B). The magnitude of the response was similar to that observed with 40 nM S-PrP. Figure 5C summarizes densitometry results obtained in 3 studies.

P3 (0.5 μM) induced PC12 cell neurite outgrowth after 48 hours, replicating the activity of S-PrP (40 nM) and nerve growth factor–β (NGF-β), as shown in the representative images in Figure 5D. The other PrP^C^-derived peptides were inactive. Image analysis of individual cells in at least 5 randomly selected fields in 3 separate experiments verified that the effects of P3 and S-PrP on neurite outgrowth were highly significant (Figure 5E).

**Figure 6:**
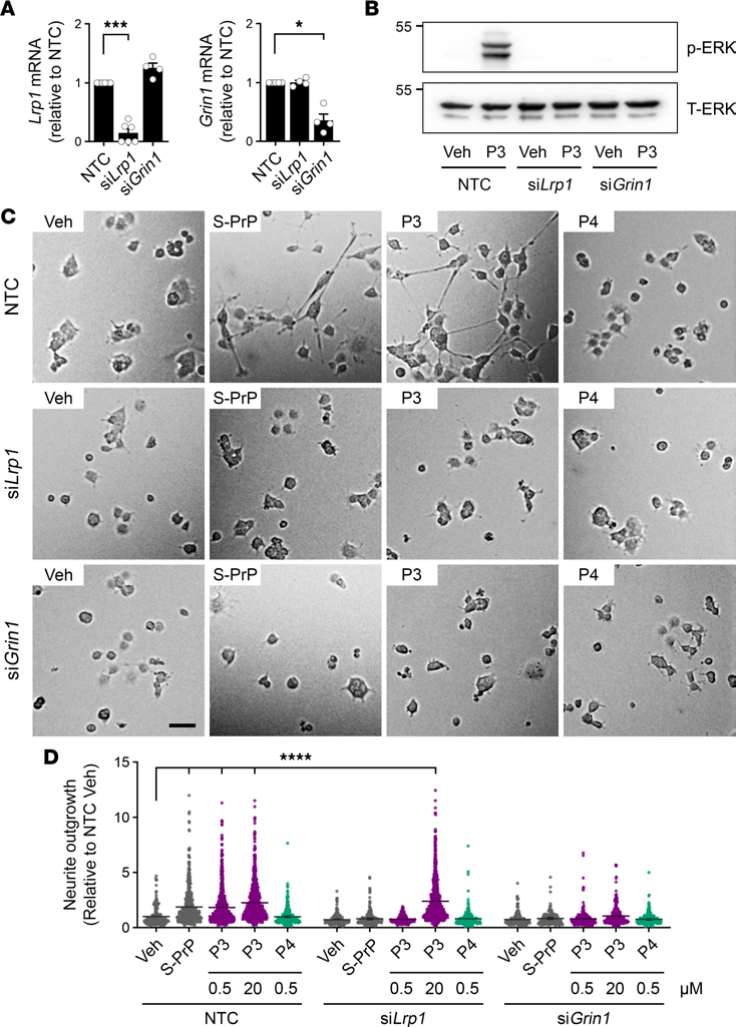
P3 activates ERK1/2 and promotes neurite outgrowth in PC12 cells by a mechanism that requires the NMDA-R and LRP1. (**A**) PC12 cells were transfected with siRNA specifically targeting *Lrp1* or *Grin1*. Control cells were transfected with NTC siRNA. Expression of the mRNAs encoding LRP1 and the GluN1 NMDA-R subunit was determined 48 hours later by RT-qPCR (*n* = 4–6; mean ± SEM; 1-way ANOVA: *P < 0.05; ***P < 0.001). (**B**) PC12 cells were transfected with *Lrp1*-specific siRNA, *Grin1*-specific siRNA, or NTC siRNA and then treated with P3 (0.5 μM) or vehicle for 10 minutes. ERK1/2 activation (p-ERK) was determined by immunoblotting. (**C**) PC12 cells were transfected with *Lrp1*-specific siRNA, *Grin1*-specific siRNA, or NTC siRNA, as indicated. The cells were then treated with S-PrP (40 nM), P3 (0.5 μM), or P4 (0.5 μM) for 48 hours. Neurite outgrowth was detected by phase contrast microscopy. Representative images are shown (scale bar, 50 μm). (**D**) Results are summarized for the studies shown in **C** and for PC12 cells treated with 20 μM P3. Neurite length was determined in all the cells of ≥5 random fields per treatment, in 3 different experiments (mean ± SEM; 1-way ANOVA: ****P < 0.0001).

To test whether LRP1 and the NMDA-R mediate the effects of P3 on cell signaling and cell physiology in PC12 cells, we silenced expression of *Lrp1* and *Grin1* in PC12 cells with siRNA. Figure 6A shows that silencing was effective; *Grin1* mRNA was not affected by *Lrp1* siRNA, and *Lrp1* mRNA was not affected by *Grin1* siRNA. P3 (0.5 μM) activated ERK1/2 in control PC12 cells transfected with nontargeting control (NTC) siRNA (Figure 6B). By contrast, P3 failed to activate ERK1/2 in PC12 cells in which *Lrp1* or *Grin1* was silenced.

Next, we studied neurite outgrowth in cells transfected with *Lrp1* siRNA, *Grin1* siRNA, or NTC siRNA. Representative images showing cells treated with P3 (0.5 μM), P4 (0.5 μM), S-PrP (40 nM), or vehicle are presented in Figure 6C. Figure 6D summarizes image analysis studies examining individual cells in at least 5 randomly selected fields from 3 separate experiments with each agonist and gene-silencing reagent. In cells transfected with NTC siRNA, neurite outgrowth was observed in response to S-PrP and P3 but not in response to P4. In cells in which *Lrp1* or *Grin1* was silenced, the response to S-PrP and 0.5 μM P3 was eliminated. Significant neurite outgrowth was observed in PC12 cells transfected with *Lrp1* siRNA and treated with 20 μM P3. By contrast, PC12 cells transfected with *Grin1* siRNA failed to respond to 20 μM P3, mimicking the results observed with BMDMs.

### P3 blocks the pro-inflammatory response of microglia to LPS.

**Figure 7:**
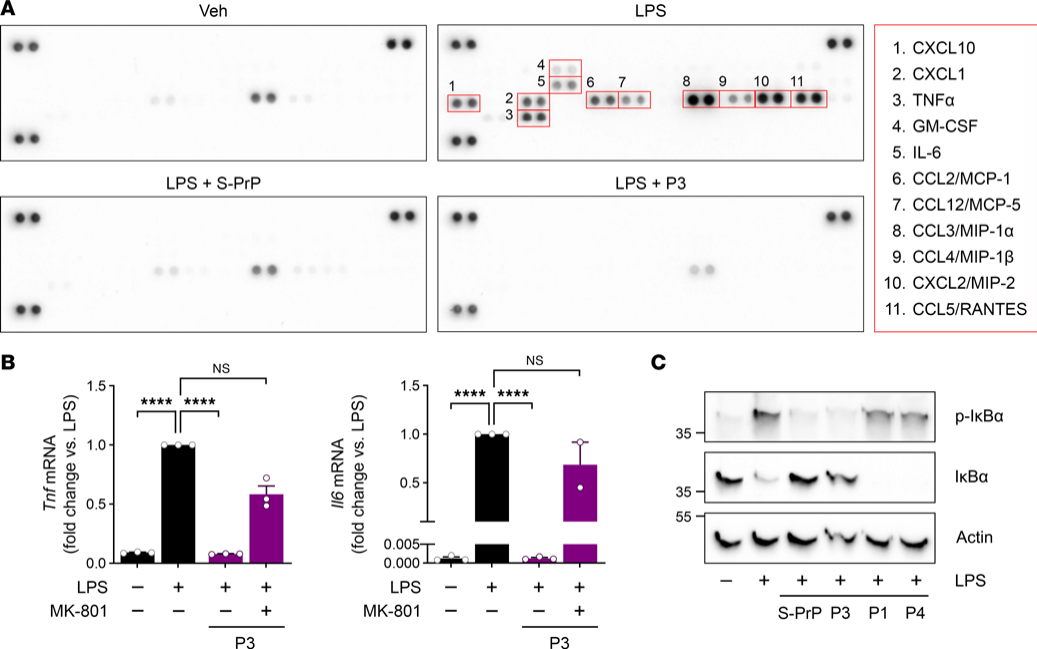
P3 inhibits the pro-inflammatory activity of LPS in microglia. (**A**) Microglia were isolated from C57BL/6J mouse pups and treated with LPS (0.1 μg/mL) for 6 hours, in the presence and absence of S-PrP (40 nM) or P3 (0.5 μM). Conditioned medium (CM) was collected and analyzed using Proteome Profiler Mouse Cytokine Array Kit (R&D Systems). Representative cytokines that were increased in CM when LPS was added in the absence of S-PrP or P3 are numbered in red boxes. (**B**) Microglia were treated with LPS (0.1 μg/mL) P3 (0.5 μM), and MK-801 (1 μM), as indicated. RT-qPCR was performed to determine mRNA levels of *Tnf* and *Il6* (mean ± SEM; *n* = 3; 1-way ANOVA: ****P < 0.0001). (**C**) Microglia were treated for 1 hour with LPS (0.1 μg/mL) in the presence or absence of S-PrP (40 nM), P1, P3, or P4 (0.5 μM). Immunoblot analysis was performed to detect p-IκBα, total IκBα, and β-actin.

Microglia are macrophage-like cells and the principal cell type responsible for innate immune responses in the CNS (44, 45). We isolated microglia from mouse pups and established primary cultures. The microglia were treated with LPS (0.1 μg/mL) for 6 hours in the presence or absence of S-PrP (40 nM) or P3 (0.5 μM). To examine cytokine production in an unbiased manner, conditioned medium (CM) was recovered and subjected to cytokine array analysis. LPS induced microglial production of multiple pro-inflammatory cytokines and chemokines, including but not limited to TNF-α, IL-6, CCL3/macrophage inflammatory protein 1α (MIP-1α), CXCL2/MIP-2, and CCL5/RANTES (Figure 7A). S-PrP inhibited cytokine expression in response to LPS, as did P3.

To validate the results of the cytokine array experiment, we performed RT-qPCR studies, examining expression of *Tnf* and *Il6*. Figure 7B shows that LPS significantly increased expression of the genes encoding both inflammatory cytokines. P3 (0.5 μM) blocked this response. When the cells were treated with the NMDA-R antagonist, MK-801, the activity of P3 was significantly inhibited, and LPS-induced pro-inflammatory cytokine expression was restored.

We also examined the ability of S-PrP and P3 to block LPS-induced IκBα phosphorylation in microglia. Figure 7C shows that S-PrP (40 nM) and P3 (0.5 μM) were effective, completely blocking IκBα phosphorylation and the associated decrease in total abundance of IκBα. P1 and P4 were ineffective.

### Lys^100^ and Lys^103^ are essential for the function of P3 as an agonist for the LRP1/NMDA-R cell-signaling receptor assembly.

**Figure 8:**
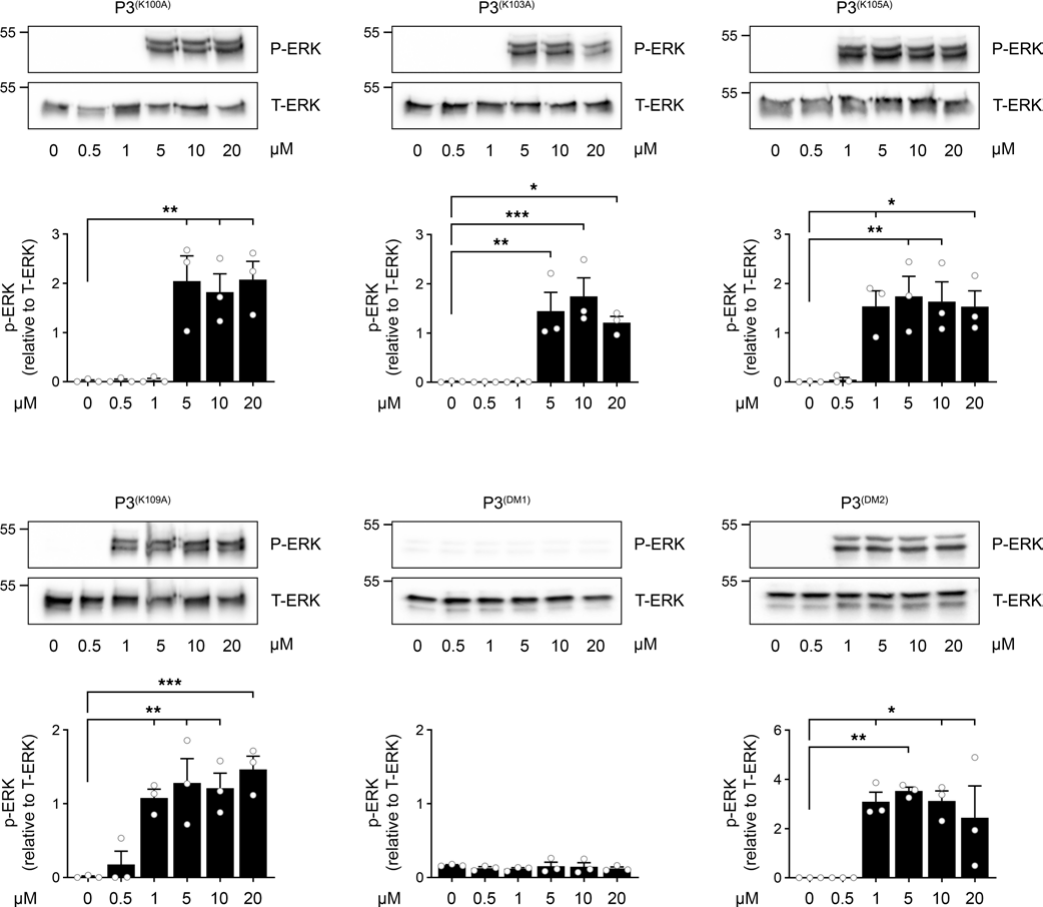
Lys^100^ and Lys^103^ are required for the function of P3 in PC12 cells. PC12 cells were treated for 10 minutes with increasing concentrations (0.5–20 μM) of P3^(K100A)^, P3^(K103A)^, P3^(K105A)^, P3^(K109A)^, P3^(DM1)^, or P3^(DM2)^. Immunoblot analysis was performed to determine ERK1/2 phosphorylation. Densitometry analysis shows p-ERK1/2 relative to total ERK1/2 (T-ERK). The bars represent the mean ± SEM of the results from 3 separate experiments (1-way ANOVA: *P < 0.05; **P < 0.01; ***P < 0.001).

Given the documented role of Lys residues in LRP1-binding motifs (26–31), we modified the 4 Lys residues in P3 to Ala, one at a time. To test the activity of the resulting set of new synthetic peptides, we began by examining ERK1/2 activation in PC12 cells. Figure 8 shows that although all 4 modified peptides demonstrated decreased potency compared with P3, peptides in which either Lys^100^ or Lys^103^ was modified to Ala demonstrated the most substantial change and were 5-fold decreased in potency compared with P3 variants in which either Lys^105^ or Lys^109^ was modified. A P3 derivative in which both Lys^100^ and Lys^103^ were modified to Ala, P3^(DM1)^, failed to activate ERK1/2 at concentrations up to 20 μM. When Lys^100^ and Lys^103^ were retained and Lys^105^ and Lys^109^ were modified to Ala, the resulting peptide, P3^(DM2)^, activated ERK1/2, and the potency was equivalent to that observed when either Lys^105^ or Lys^109^ was modified individually.

**Figure 9:**
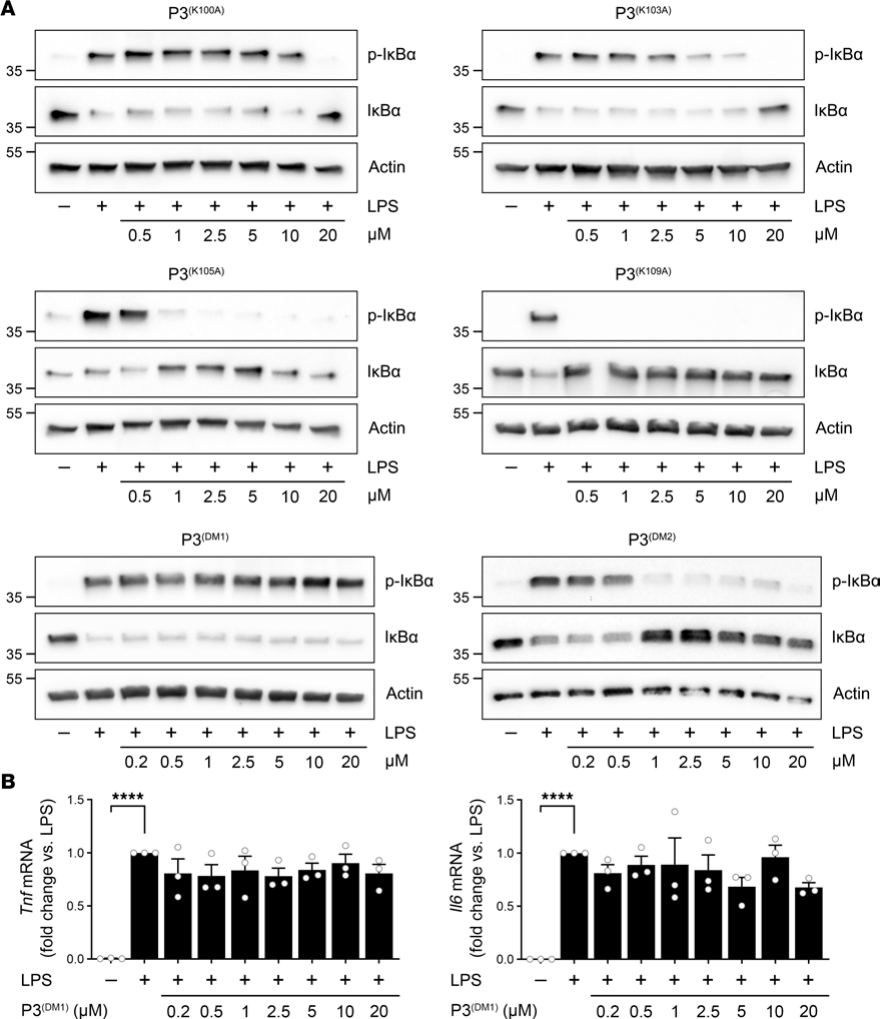
Lys^100^ and Lys^103^ are required for the function of P3 in macrophages. (**A**) BMDMs from wild-type mice were treated for 1 hour with LPS (0.1 μg/mL) and increasing concentrations (0.2–20 μM) of P3^(K100A)^, P3^(K103A)^, P3^(K105A)^, P3^(K109A)^, P3^(DM1)^, or P3^(DM2)^, as indicated above each panel. Immunoblot analysis was performed to detect p-IκBα, total IκBα, and β-actin. (**B**) BMDMs from wild-type mice were treated for 6 hours with LPS (0.1 μg/mL) in the presence of increasing concentrations of P3^(DM1)^ (0.2–20 μM). RT-qPCR was performed to determine mRNA levels for *Tnf* and *Il6* (mean ± SEM; *n* = 3; 1-way ANOVA: ****P < 0.0001).

Next, we examined the ability of modified P3 peptides to inhibit LPS-induced NF-κB activation in BMDMs. Cells were treated with 0.1 μg/mL LPS and with the indicated concentrations of peptide for 1 hour. Representative blots showing p-IκBα and total IκBα are shown in Figure 9A. Densitometry results summarizing the results of 3 separate experiments with each peptide are shown in Supplemental Figure 1; supplemental material available online with this article; https://doi.org/10.1172/jci.insight.170121DS1 P3^(K105A)^ and P3^(K109A)^ were only slightly less active than unmodified P3. By contrast, P3^(K100A)^ and P3^(K103A)^ demonstrated substantially decreased potency compared with P3 and completely inhibited LPS-induced IκBα phosphorylation only when present at 20 μM. P3^(DM1)^ was ineffective throughout the concentration range studied, whereas P3^(DM2)^ retained activity.

To verify that P3^(DM1)^ is ineffective at opposing the response to LPS in BMDMs, we examined *Tnf* mRNA expression in cells treated for 6 hours with LPS and with the indicated concentrations of P3^(DM1)^ (Figure 9B). P3^(DM1)^ failed to inhibit LPS-induced *Tnf* mRNA expression throughout the studied P3^(DM1)^ concentration range. Similarly, P3^(DM1)^ failed to inhibit LPS-induced *Il6* mRNA expression.

### P3 rescues the phenotype of Prnp^–/–^ mice in LPS challenge experiments.

**Figure 10:**
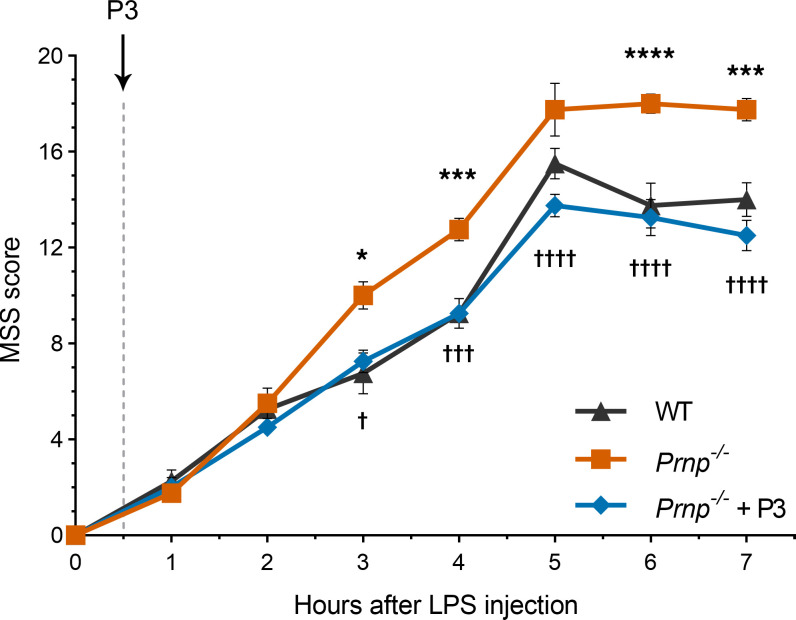
P3 rescues the increased susceptibility of *Prnp*^–/–^ mice to LPS. Male 16- to 20-week old *Prnp*^–/–^ mice (shown in orange) and wild-type mice in the same genetic background (shown in black) were challenged with LPS, by IP injection, at 75% of the LD_50_. A second matched cohort of *Prnp*^–/–^ mice was treated with LPS and then with P3, 0.5 hour later (blue). Toxicity was scored as described in Methods. *Prnp*^–/–^ mice demonstrated significantly more toxicity compared with wild-type mice (mean ± SEM; *n* = 4; 2-way ANOVA: *P < 0.05; ***P < 0.001; ****P < 0.0001). P3 significantly reversed the toxicity of LPS in *Prnp*^–/–^ mice (mean ± SEM; *n* = 4; 2-way ANOVA: ^†^*P* < 0.05; ^†††^*P* < 0.001; ^††††^*P* < 0.0001). MSS, mouse sepsis score.

We performed experiments to test whether we can replicate the reported increase in sensitivity of *Prnp*^–/–^ mice to LPS challenge (46). These experiments were performed as previously described (22, 42), using the *Prnp*^ZH3/ZH3^ strain (47). Male *Prnp*^–/–^ mice and wild-type mice in the same genetic background (26–28 g) were challenged with LPS at 75% of the LD_50_ calculated for wild-type mice. Animals were monitored and scored for signs of toxicity using the murine sepsis scoring system (48). Figure 10 shows that *Prnp*^–/–^ mice demonstrated significantly increased sensitivity to LPS, compared with wild-type mice. When *Prnp*^–/–^ mice were injected intravenously with a single dose of P3 (2.5 μg/g body weight), 30 minutes after LPS administration, toxicity was significantly decreased.

## Discussion

PrP^C^ has been identified as a gene product capable of attenuating inflammation in a variety of contexts (33–38, 46, 49–53), including experimental autoimmune encephalitis (33–35) and ischemic brain injury (33, 36–38). Our prior work identified PrP^C^ derivatives released by cells, including soluble fragments of PrP^C^ and EV-associated PrP^C^, as candidate mediators of the known antiinflammatory activity of PrP^C^ (22, 25). We also implicated LRP1 and the NMDA-R as cell-signaling receptors for soluble- and EV-associated PrP^C^ derivatives. PrP^C^ that localizes to lipid rafts, within the original cell of synthesis, also may express LRP1-dependent antiinflammatory activity by laterally associating with LRP1 within the plasma membrane; this interaction facilitates the antiinflammatory activity of LRP1, when it is presented with ligands other than S-PrP, such as tissue-type plasminogen activator (tPA) (25).

The studies presented here support our model in which PrP^C^ derivatives released from cells function as LRP1-dependent cell-signaling agonists and antiinflammatory agents. We demonstrated that S-PrP blocks inflammatory responses in microglia, supporting the hypothesis that the PrP^C^/LRP1 interaction may be responsible for the documented antiinflammatory activity of PrP^C^ in the CNS (33–38). We also harnessed the cell-signaling and antiinflammatory activity of PrP^C^ within a single 14-mer peptide, derived from the structure of PrP^C^. This advance suggests that it is feasible to translate the known antiinflammatory activities of PrP^C^ into novel small molecule candidate therapeutics.

The ability of a small peptide to mimic the cell-signaling and antiinflammatory activities of full-length S-PrP was not anticipated. LRP1 ligands that activate antiinflammatory cell-signaling pathways share a common mechanism of receptor engagement, in which at least 2 receptors, LRP1 and the NMDA-R, play an instrumental role (22, 25, 42). The NMDA-R appears to be essential. LRP1 substantially decreases the concentration of ligand required to trigger cell signaling. Thus, it is reasonable to propose that LRP1 captures soluble ligands, like S-PrP, then delivers them to the NMDA-R to trigger calcium influx and activation of cell-signaling factors such as Src family kinases and PI3K. Notably, the NMDA-R is reported to bind tPA and PrP^C^ independently of LRP1 (54–56).

If LRP1 transfers antiinflammatory ligands to the NMDA-R, the ligand would most likely form a transient ternary complex in which different regions of the ligand engage LRP1 and the NMDA-R simultaneously. Such a model seems highly feasible for tPA, which has multiple domains (57), and for α_2_M, which is a large tetramer of 4 identical subunits (58). The size of P3, a synthetic 14–amino acid peptide, argues against the bridged receptor model. Tandem Lys residues in the structure of P3, including Lys^100^ and Lys^103^, were essential for activation of cell signaling via the LRP1/NMDA-R assembly. Replacement of both Lys residues with Ala in P3^(DM1)^ eliminated activity. Tandem Lys residues have been implicated in the binding of a number of full-length proteins to LRP1 (26–31), though the activity of the Lys residues in LRP1/NMDA-R–dependent cell signaling has not been formally addressed in previous studies to our knowledge. Although it is unlikely that P3 bridges LRP1 to the NMDA-R, both receptors were necessary to elicit potent P3 biological activities.

In addition to its activity in cell culture model systems, P3 rescued the known increase in susceptibility of *Prnp*^–/–^ mice to LPS challenge. This result has a number of implications. First, these studies suggest that overly exuberant pro-inflammatory responses in *Prnp*^–/–^ mice may be rescued entirely by soluble derivatives of PrP^C^. Second, although we did not study the pharmacokinetics of P3, synthetic peptides typically have a short circulating half-life (32). Assuming an initial distribution volume corresponding to the plasma volume in a mouse (1.5 mL) and the molecular mass of P3 of 1,743, the maximum concentration of P3 in the plasma following injection was estimated at about 50 μM. We hypothesize that P3 rapidly engages cellular receptor targets and stimulates changes in cell physiology that are long-lasting in vivo, despite clearance of the peptide. In support of this hypothesis, we previously demonstrated that a single intravenously administered injection of enzymatically inactive tPA not only neutralizes LPS toxicity but also significantly reverses inflammation and disease progression in the dextran sodium sulfate model of inflammatory bowel disease (42, 59). These results are observed despite the fact that the circulating half-life of inactive tPA in mice is only 3 minutes (60).

The PrP^C^-specific monoclonal antibody, POM2, blocked the ability of P3 to inhibit LPS-induced IκBα phosphorylation; POM1, which targets a separate region of the PrP^C^ structure, was ineffective. In the POM series of PrP^C^-specific monoclonal antibodies studied by us, POM2 is the only antibody that blocks the effects of both S-PrP and EV-associated PrP^C^ on cell signaling and cell physiology (21–23). Epitope mapping has shown that POM2 recognizes the octarepeat region of PrP^C^, which is N-terminal to P3 (43). POM3, which recognizes an epitope between the octarepeat region and P3 (43), also has been studied by us and is inactive in disrupting LRP1-dependent cell signaling by S-PrP and EV-associated PrP^C^ (21–23). Because the activity of S-PrP is unaltered in PrP^C^-deficient cells (21–23), we have assumed that POM2 targets the ligand, S-PrP or EV-associated PrP^C^, and does not target cell PrP^C^. However, there is considerable evidence that PrP^C^ associates with the LRP1/NMDA-R complex in lipid rafts and is involved in LRP1-initiated cell signaling (20, 25). Thus, although *Prnp* gene deletion or gene silencing has no effect on cell signaling triggered by released forms of PrP^C^, in target cells that express PrP^C^, POM2 may disrupt interaction of soluble PrP^C^ derivatives with the LRP1/NMDA-R assembly.

In addition to membrane-anchored PrP^C^, the LRP1/NMDA-R assembly may associate with other proteins to trigger cell signaling. For example, the endoplasmic reticulum chaperone, Grp78, associates with LRP1 when released by cells and may participate in activation of cell signaling via the LRP1/NMDA-R assembly (61, 62). Other receptors that interact with PrP^C^ to mediate cell-signaling events include NCAM, mGluR5, and Adgrg6/gpr126 (63–65). Whether these receptors function independently or in concert with the LRP1/NMDA-R assembly is not currently understood. Variable association of other cell-signaling receptors with the LRP1/NMDA-R assembly provides a hypothetical mechanism by which the response to various ligands may be cell type specific (57).

Identification of the putative LRP1-binding motif in PrP^C^ allows us to speculate regarding the role of the LRP1/NMDA-R assembly in previously identified PrP^C^-mediated events. Guillot-Sestier et al. (66) demonstrated that a proteolytically released PrP^C^ fragment, referred to as N1, demonstrates neuroprotective activity by modulating the p53 pathway. N1 includes residues 23–110 and, thus, the LRP1-binding motif in P3. It is thus reasonable to speculate that N1 is an LRP1 ligand. Like N1, LRP1 ligands are reported to activate cell-signaling pathways that are neuroprotective (67). Similarly, the region of PrP^C^ implicated in Schwann cell signaling via Adgrg6/gpr126 (65) includes the P3 LRP1-binding motif. However, Küffer et al. (65) provided evidence suggesting that the Lys-rich N-terminus of PrP^C^ is required for activation of cell signaling via gpr126. In our study, the N-terminus of PrP^C^ was contained within P1, which was inactive against macrophages and PC12 cells.

Finally, our results demonstrating the ability of S-PrP and P3 to inhibit pro-inflammatory cytokine expression by microglia suggest a role for the PrP^C^-LRP1/NMDA-R pathway in the regulation of neuro-inflammation and neurodegenerative diseases. Proteins implicated in neurodegeneration, including amyloid-β, microtubule-associated protein tau, and α-synuclein are known to activate microglia, which may contribute to disease progression (68–70). Understanding whether binding of PrP^C^ derivatives to microglial LRP1 regulates this process is an important future goal.

## Methods

### Proteins and reagents.

S-PrP (residues 23–231 from mouse PrP^C^) was expressed and purified as previously described (21) and provided by Christina Sigurdson (University of California San Diego, La Jolla, California, USA). Peptides P1, P2, P3, P3*, P4, P3^(K100A)^, P3^(K103A)^, P3^(K105A)^, P3^(K109A)^, P3^(DM1)^, and P3^(DM2)^ were provided by AnaSpec. All peptides had N-terminal acetylation and C-terminal amidation. LPS serotype 055:B5 from *E*. *coli* was from MilliporeSigma. The uncompetitive NMDA-R antagonist, dizocilpine (MK-801), was from Cayman Chemical. Recombinant human NGF-β was from R&D Systems. The PrP^C^-specific monoclonal antibodies, POM1 are POM2, were previously described (43).

### Animals.

Wild-type C57BL/6J mice were obtained from The Jackson Laboratory. To generate mice in which BMDMs are LRP1 deficient, *Lrp1*^fl/fl^ mice were bred with mice that express Cre recombinase under the control of the LysM promoter (LysM-Cre), in the C57BL/6J background, as previously described (42). To generate mice in which macrophages are deficient in the essential NMDA-R GluN1 subunit, *Grin1*^fl/fl^ mice were bred with mice that express Cre recombinase under the control of the LysM-Cre promoter in the C57BL/6J background. Control cells were harvested from littermates that were *Grin1*^fl/fl^ but LysM-Cre^–^. *Prnp*^−/−^ mice were provided by Adriano Aguzzi (University Hospital of Zurich, Zurich, Switzerland).

### Cell culture model systems.

BMDMs were harvested from 16-week-old wild-type male mice, as previously described (40, 42). Briefly, bone marrow cells were flushed from mouse femurs, plated in dishes that were not tissue culture treated, and cultured in DMEM/F-12 medium containing 10% FBS and 20 nM mouse macrophage colony-stimulating factor (BioLegend) for 7 days. Nonadherent cells were eliminated. Adherent cells included more than 95% BMDMs, as determined by F4/80 and CD11b immunoreactivity. This method was approved by the IACUC of the University of California San Diego (UCSD).

Rat PC12 cells were from the ATCC (CRL-1721) and subjected to quality control tests by the ATCC. PC12 cells were cultured in DMEM, high glucose (Thermo Fisher Scientific), containing 10% heat-inactivated FBS, as well as 5% heat-inactivated horse serum (Thermo Fisher Scientific), in plates that were precoated with 0.01 mg/mL type IV collagen (MilliporeSigma). Cells were passaged no more than 8 times.

Microglia were isolated from C57BL/6J mouse pups, as described previously (71). In brief, brains were harvested from postnatal day 1–6 mice. The cortices were dissected from the forebrain, and the surrounding meninges were removed. Intact cortices were mechanically and enzymatically dissociated using the Neural Tissue Dissociation Kit (Miltenyi Biotec). Mixed glial cultures were established in DMEM/F-12 supplemented with GlutaMAX (Thermo Fisher Scientific), 10% FBS, and 1× Gibco Antibiotic-Antimycotic (Thermo Fisher Scientific). After culturing 10–14 days, microglia were harvested by shaking the mixed cultures at 200 rpm for 30 minutes at 37°C. The floating cells were collected by centrifugation (5 minutes, 600*g*) and replated at 3 × 10^5^ cells/well. Culture purity was more than 96% as determined by immunofluorescence microscopy for Iba1 (positive), glial fibrillary acidic protein (negative), βIII-tubulin (negative), and OLIG1 (negative). Experiments were performed within 24 hours of completing cell isolations.

### Gene silencing.

Rat-specific ON-TARGETplus SMARTpool siRNA, targeting *Lrp1* or *Grin1*, and pooled NTC siRNA were from Horizon Discovery. PC12 cells (2 × 10^6^) were transfected with siRNA by electroporation using the Cell Line Nucleofector Kit V (Lonza), following the manufacturer’s instructions. Briefly, cell suspensions were treated with 300 nM *Lrp1*-specific siRNA, *Grin1*-specific siRNA, or NTC siRNA, then electroporated with the PC12-specific program in a Lonza Nucleofector 2b device. Gene silencing was determined 48 hours after transfection by RT-qPCR, as previously described (23). Experiments were performed 48 hours after transfection.

### Gene expression studies.

BMDMs were transferred to serum-free medium (SFM) for 30 minutes and treated for 6 hours with various proteins and reagents, including LPS (0.1 μg/mL), various synthetic peptides at different concentrations, MK-801 (1 μM), or vehicle (20 mM sodium phosphate, 150 mM NaCl, pH 7.4, with PBS). RNA was isolated using the NucleoSpin RNA kit (Macherey-Nagel) and reverse-transcribed using the iScript cDNA synthesis kit (Bio-Rad). qPCR was performed using TaqMan gene expression products (Thermo Fisher Scientific). Primer-probe sets were as follows: *Gapdh* (Mm99999915_g1), *Tnf* (Mm00443258_m1), and *Il6* (Mm00446190_m1). The relative change in mRNA expression was calculated using the 2^ΔΔCt^ method with *Gapdh* mRNA as a normalizer.

### Flow cytometry.

The abundance of the NMDA-R on the surfaces of BMDMs was determined by flow cytometry. Nonpermeabilized cells were labeled with NMDA-R GluN1 subunit-specific antibody (catalog PA3-102, Invitrogen, Thermo Fisher Scientific). Cell-associated PA3-102 was detected with Alexa Fluor 647–conjugated secondary antibody (catalog A21244, Invitrogen, Thermo Fisher Scientific). Control cells were treated with secondary antibody only. All data were analyzed using FlowJo Software version 10.7.1 (BD Biosciences).

### Cell signaling.

BMDMs were transferred to SFM for 30 minutes and treated for 1 hour with various proteins and reagents, alone or simultaneously as noted. PC12 cells were cultured in serum-containing medium until approximately 70% confluent. The cells were then transferred into SFM for 2 hours before treatment with various reagents. Some cultures were pretreated with MK-801 (1 μM), as noted. Microglia were cultured in SFM for 30 minutes and then treated with LPS (0.1 μg/mL) for 1 hour in the presence and absence of S-PrP (40 nM) or synthetic peptides.

Extracts of BMDMs, PC12 cells, and microglia were prepared in RIPA buffer (20 mM sodium phosphate, 150 mM NaCl, pH 7.4, 1% Triton X-100, 0.5% sodium deoxycholate, 0.1% SDS) supplemented with protease and phosphatase inhibitors (Thermo Fisher Scientific). Equal amounts of protein were subjected to SDS-PAGE and electro-transferred to PVDF membranes. The membranes were blocked with 5% nonfat dried milk and then incubated with primary antibodies from Cell Signaling Technology that recognize p-ERK1/2 (catalog 9102), total ERK1/2 (catalog 4370), p-IκBα (catalog 2859), total IκBα (catalog 9242), and β-actin (catalog 3700). The membranes were washed and incubated with horseradish peroxidase–conjugated secondary antibodies from Jackson ImmunoResearch (anti-rabbit: catalog 111-035-003; anti-mouse: catalog 115-035-003). Immunoblots were developed using Thermo Fisher Scientific SuperSignal West Pico PLUS Chemiluminescent Substrate and imaged using the Azure Biosystems c300 digital system. Images were processed with Adobe Photoshop 23.3.2. When immunoblots were reprobed, p-IκBα was detected first, followed by total IκBα, and then, β-actin. Presented results are representative of at least 3 independent experiments.

### PC12 cell neurite outgrowth.

Wild-type PC12 cells and cells transfected with *Lrp1*-specific, *Grin1*-specific, or NTC siRNA were plated at 1 × 10^5^ cells/well and maintained in serum-containing medium for 24 hours. The medium was then replaced with SFM supplemented with S-PrP (40 nm), synthetic peptides (0.5 or 20 μM), or NGF-β. Incubations were conducted for 48 hours. The cells were imaged by phase contrast microscopy, using a Leica DMi8 microscope equipped with a Leica DFC3000 G digital camera and Leica Application Suite X software. Neurite length was determined in all the cells imaged in at least 5 representative fields in 3 separate experiments using the NeuronJ plugin of ImageJ software (NIH).

### Proteome profiler mouse cytokine array.

Microglia were transferred to SFM for 30 minutes and treated with LPS (0.1 μg/mL) in the presence and absence of S-PrP (40 nM) or P3 (0.5 μM) for 6 hours. CM was collected and particulates were removed by centrifugation at 800*g*. An equivalent amount of CM (1.0 mL for each condition) was incubated with the nitrocellulose membranes provided in the Proteome Profiler Mouse Cytokine Array Kit (R&D Systems). Membranes were developed following the instructions of the manufacturer.

### LPS challenge experiments in Prnp^–/–^ mice.

Male *Prnp*^–/–^ mice and wild-type mice in the same genetic background (16–20 weeks old, 26–28 g) were injected intraperitoneally with 9 mg/kg LPS. The LD_50_ for the specific LPS lot was predetermined in our laboratory, as previously described by us (42), and was 12 mg/kg. The mice were treated by intravenous injection with P3 (2.5 μg/g body weight) or PBS, 30 minutes after LPS administration. Animals were monitored and scored for signs of toxicity at 1-hour intervals using the murine sepsis scoring system (48). The following variables were scored from 0 to 4: appearance, level of consciousness, activity, responses to auditory stimuli, eye function, respiration rate, and respiration quality. Mice were considered moribund and euthanized if the murine sepsis score was 21 or higher. Investigators were masked to treatment groups.

### Statistics.

Statistical analysis was performed using GraphPad Prism 9.4. All results are expressed as the mean ± SEM. Each replicate was performed using a different BMDM or PC12 cell preparation. Comparisons between 2 groups were performed using 2-tailed unpaired *t* tests. When more than 2 groups were compared, we performed 1-way ANOVA followed by post hoc Dunnett’s multiple-comparison test. LPS challenge experiments were analyzed by 2-way ANOVA followed by Holm-Šidák multiple-comparison test. *P* < 0.05 was considered statistically significant.

### Study approval.

All animal experiments were approved by the IACUC of UCSD and were conducted strictly under the guidelines for animal experimentation of UCSD.

### Data availability.

The uncropped images of original immunoblot membranes are available from the corresponding author. Values for all data points in graphs can be found in the Supporting Data Values file.

## Author contributions

SLG conceived of the idea; EM, PA, and SLG designed the experiments; EM, PA, CBG, and CZ conducted the experiments; KRH completed the PC12 cell neurite outgrowth image analysis; all authors contributed to data interpretation; EM and SLG wrote the first draft of the manuscript; and all authors read and approved the final draft of the paper.

## Supplementary Material

Supplemental data

Supporting data values

## Version 1 06/27/2023

In-Press Preview

## Version 2 08/08/2023

Electronic publication
