# Supplementary material for: An LRP1-binding motif in cellular prion protein replicates cell-signaling activities of the full-length protein
Source: JCI Insight. 2023 Aug 8;8(15):e170121. doi: 10.1172/jci.insight.170121 (PMC10445690; doi:10.1172/jci.insight.170121)
Supplement: Supplemental data [file jciinsight-8-170121-s022.pdf]

Figure S1

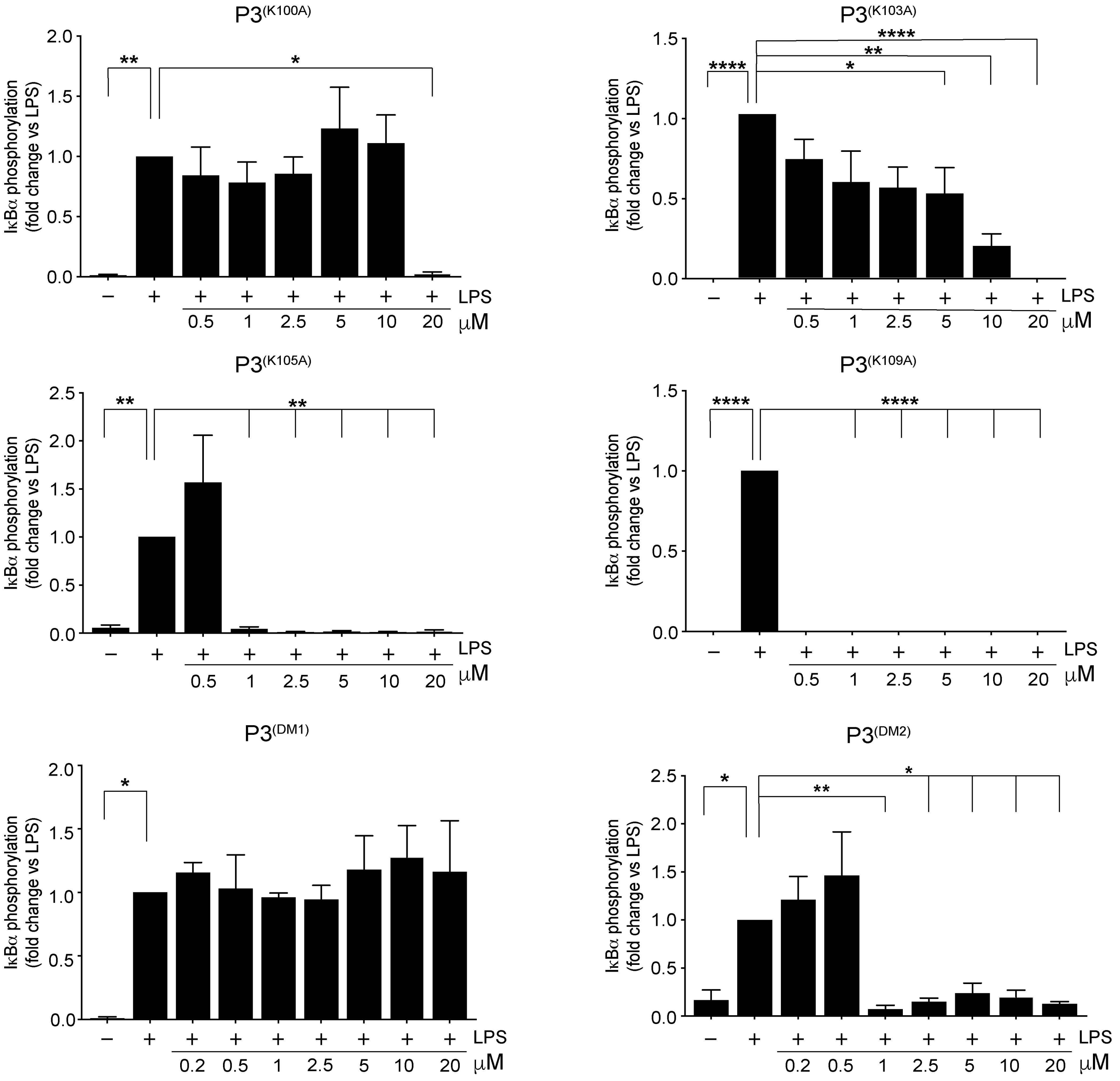

Supplementary Figure 1. Densitometry analysis of phospho-IκBα relative to actin in immunoblots of BMDMs treated with LPS and increasing concentrations of the six P3 peptide derivatives. The results of three separate studies are summarized (mean ± SEM; one-way ANOVA: *P<0.05; **P<0.01; ****P<0.0001).
